# Lack of Association of miR-146a rs2910164 Polymorphism with Gastrointestinal Cancers: Evidence from 10206 Subjects

**DOI:** 10.1371/journal.pone.0039623

**Published:** 2012-06-27

**Authors:** Fang Wang, Guoping Sun, Yanfeng Zou, Lulu Fan, Bing Song

**Affiliations:** 1 Department of Oncology, The First Affiliated Hospital of Anhui Medical University, Hefei, Anhui, China; 2 Department of Epidemiology and Biostatistics, School of Public Health, Anhui Medical University, Hefei, Anhui, China; 3 Department of Cardiology, The First Affiliated Hospital of Anhui Medical University, Hefei, Anhui, China; University of Chicago, United States of America

## Abstract

**Background:**

Recent studies on the association between miR-146a rs2910164 polymorphism and risk of gastrointestinal (GI) cancers showed inconclusive results. Accordingly, we conducted a comprehensive literature search and a meta-analysis to clarify the association.

**Methodology/Principal Findings:**

Data were collected from the following electronic databases: Pubmed, Excerpta Medica Database (Embase), and Chinese Biomedical Literature Database (CBM), with the last report up to February 24, 2012. The odds ratio (OR) and its 95% confidence interval (95%CI) were used to assess the strength of association. Ultimately, a total of 12 studies (4,817 cases and 5,389 controls) were found to be eligible for meta-analysis. We summarized the data on the association between miR-146a rs2910164 polymorphism and risk of GI cancers in the overall population, and performed subgroup analyses by ethnicity, cancer types, and quality of studies. In the overall analysis, there was no evidence of association between miR-146a rs2910164 polymorphism and the risk of GI cancers (G versus C: OR = 1.07, 95%CI 0.98−1.16, *P* = 0.14; GG+GC versus CC: OR = 1.14, 95%CI 1.00−1.31, *P* = 0.05; GG versus GC+CC: OR = 1.06, 95%CI 0.91−1.23, *P* = 0.47; GG versus CC: OR = 1.17, 95%CI 0.95−1.44, *P* = 0.13; GC versus CC: OR = 1.14, 95%CI 1.00−1.31, *P* = 0.05). Similar results were found in the subgroup analyses by ethnicity, cancer types, and quality of studies.

**Conclusions/Significance:**

This meta-analysis demonstrates that miR-146a rs2910164 polymorphism is not associated with GI cancers susceptibility. More well-designed studies based on larger sample sizes and homogeneous cancer patients are needed.

## Introduction

MicroRNAs (miRNAs) are small, non-coding, endogenous RNAs that represent a significant mechanism of post transcriptional gene regulation [1]. It has been demonstrated that miRNAs have a crucial function in affecting processes as varied as cellular differentiation, proliferation, metabolism, apoptosis, and tumorigenesis [2]. Several miRNA expression analyses in human epithelial malignancies have shown that distinct tumor specific miRNA signatures can distinguish different cancer types and classify their sub-types [3]. Some of the key dysregulated miRNAs have the potential value to be molecular bio-markers, which can improve diagnosis, prognosis, and monitoring of treatment response for human cancers [4]–[6]. Some miRNAs may function as oncogenes or tumor suppressors [7]–[8].

Primary miRNA transcripts are cleaved by Ribonuclease (RNase) III Drosha in the cell nucleus into 70-nucleotide to 80-nucleotide precursor miRNA (pre-miRNA) hairpins and transported to the cytoplasm. Then, pre-miRNAs are processed by RNase III Dicer into miRNA: miRNA duplexes. One strand of these duplexes is generally degraded, whereas the other is used as mature miRNA. Mature miRNAs can recognize and bind into the 3′-untranslated region (UTR) of the target mRNAs and interfere with their translation [9]. It was hypothesized that single nucleotide polymorphisms (SNPs) within the miRNA sequence or miRNA target could either weaken or reinforce the binding between miRNA and target [10].

In the human genome, miR-146a is located at chromosome 5q33. Many recent studies have suggested that miR-146a expression is deregulated in many solid tumors [11]–[14]. It became evident that miR-146a may act as a tumor suppressor. So far, the possible mechanism through which a miR-146a downmodulation may contribute to tumor development remains unclear. However, it seems related to the capacity of this miRNA to target some mRNAs [15]. A G>C polymorphism has been identified in the miR-146a gene, and the reference number of this SNP in the database of the National Center for Biotechnology information (NCBI) is rs2910164 [16].This polymorphism exists in the stem region opposite to the mature miR-146a sequence, which leads to a change from G:U pair to C:U mismatch in the stem structure of miR-146a precursor [16].A recent study provided evidence that the existence of a common G/C polymorphism within the pre-miR-146a sequence reduced production of miR-146a [17]. This may lead to a reduced downmodulation of the corresponding target genes. Several studies have reported that this polymorphism could contribute to tumorigenesis of many cancers, especially those belonging to the gastrointestinal (GI) tract, such as gastric cancer and hepatocellular carcinoma [18]–[19].

The GI cancers share a number of characteristics that suggest common etiological pathways or mechanisms. Thus, the identification of possible risk factors and carcinogenetic mechanisms is essential for the prevention of these cancers. Accumulating evidence has uncovered the important role of inflammatory network in the promotion of GI cancer development [20]. Regular use of non-steroidal anti-inflammatory drugs (NSAIDs) could lower the mortality rate from cancers in the gastrointestinal tract [21]. Environmental, dietary and endogenous risk factors are thought to exert important effects on individual predisposition [10]. From 2008 to 2012, researchers have consecutively reported associations between miR-146a rs2910164 polymorphism and risk of GI cancers, but with mixed or even conflicting results [22]–[33]. Accordingly, we aimed to conduct a meta-analysis to shed more light on the role of miR-146a rs2910164 polymorphism in susceptibility to GI cancers.

## Materials and Methods

### Identification of Eligible Studies

We performed a systematic search using Pubmed, Excerpta Medica Database (Embase) and Chinese Biomedical Literature Database (CBM) with the last search updated on February 24, 2012. The following search terms were used: “microRNA OR mir OR miRNA”, “cancer OR carcinoma OR adenocarcinoma OR neoplasm OR tumour OR tumor”, “gene OR polymorphism OR allele OR variation”, and “146a OR rs2910164”. Searching was done without restriction on language or publication years. We evaluated all associated publications to retrieve the most eligible literature. Their reference lists were searched manually to identify additional eligible studies.

### Inclusion and Exclusion Criteria

The following inclusion criteria were used in selecting literature for further meta-analysis: (1) evaluation of miR-146a rs2910164 polymorphism and GI cancers; (2) independent case-control studies for human; (3) describing useful genotype frequencies; (4) only full-text manuscripts were included. Exclusion criteria included: (1) duplication of the previous publications; (2) abstract, comment, review and editorial; (3) family-based studies of pedigrees with several affected cases per family. When a study reported the results on different ethnicities, we treated them as separate studies. When there were multiple publications from the same population, only the largest study was included.

### Data Extraction

Two investigators independently extracted the data according to the inclusion criteria listed above. Discrepancies were adjudicated by a third investigator until consensus was achieved on every item. The following information was extracted from each eligible study using a standardized data collection protocol (the PRISMA checklist, Table S1): the first author’s name, year of publication, source of publication, ethnicity, cancer types, definition and numbers of cases and controls, and allele as well as genotype frequencies for cases and controls. If original genotype frequency data was unavailable in relevant articles, a request for additional data was sent to the corresponding author.

### Quality Score Assessment

The quality of the studies was independently assessed by two investigators according to a set of predetermined criteria which was extracted and modified from previous studies [34]–[35] (Table 1). These scores were based on traditional epidemiological considerations, as well as cancer genetic issues. Any disagreement was resolved by discussion between the two investigators. Scores ranged from the lowest zero to the highest 18. Articles scoring <12 were classified as “low quality”, and those ≥12 as “high quality”.

**Table 1:** Scale for quality assessment.

Criterion	Score
*Source of cases*	
Selected from population or cancer registry	3
Selected from hospital	2
Selected from pathology archives, but without description	1
Not described	0
*Source of controls*	
Population-based	3
Blood donors or volunteers	2
Hospital-based (cancer-free patients)	1
Not described	0
*Case-control match*	
Matched by age and gender	3
Not matched by age and gender	0
*Specimens used for determining genotypes*	
White blood cells or normal tissues	3
Tumor tissues or exfoliated cells of tissue	0
*Hardy-Weinberg equilibrium in controls*	
Hardy-Weinberg equilibrium	3
Hardy-Weinberg disequilibrium	0
*Total sample size*	
>1000	3
>500 and <1000	2
>200 and <500	1
<200	0

### Meta-analysis Methods

We used the PRISMA checklist as protocol of the meta-analysis and followed the guideline (Table S1) [36]. We first assessed Hardy-Weinberg equilibrium for each study using Chi-square test in control groups. The odds ratio (OR) and its 95% confidence interval (95%CI) were used to assess the strength of the association between miR-146a rs2910164 polymorphism and risk of GI cancers based on genotype frequencies in cases and controls. The pooled ORs were performed for allelic comparison (G versus C), dominant model (GG + GC versus CC), recessive model (GG versus GC+CC), homozygote comparison (GG versus CC) and heterozygote comparison (GC versus CC), respectively. The significance of the pooled OR was determined by the *Z*-test. Stratified analyses were performed by ethnicity, cancer types and quality of studies. A Chi-square test based Q-statistic was performed to assess the between-study heterogeneity [37]. If the heterogeneity was not significant, the fixed effect model (using the Mantel-Haenszel method) was used to estimate the summary OR and 95% CI; otherwise, the random effect model (using the DerSimonian and Laird method) was used [38]–[39]. We also measured the effect of heterogeneity by another measure, *I^2^* = 100%×(Q-df)/Q [40].

### Evaluation of Publication Bias

Potential publication bias was estimated using Egger’s linear regression test (*P*<0.05 was considered significant) by visual inspection of the funnel plot [41]. Analyses were performed using the software Review Manager 4.2 (Cochrane Collaboration, http://www.cc-ims.net/RevMan/relnotes.htm/) and Stata version 10 (StataCorp LP, College Station, Texas, USA). The pooled ORs were performed for allelic comparison, dominant model, recessive model, homozygote comparison and heterozygote comparison, respectively. Thus, the Bonferroni method was used to adjust the significance alpha level to correct for the problem of multiple comparisons. Specifically, the usual significance level (α = 0.05) was divided by 5 to account for five comparisons. Thus, a *P* value less than 0.01 was considered statistically significant in the study, and all the *P* values were two sided.

## Results

### Characteristics of Eligible Studies (Table 2)

**Figure 1:**
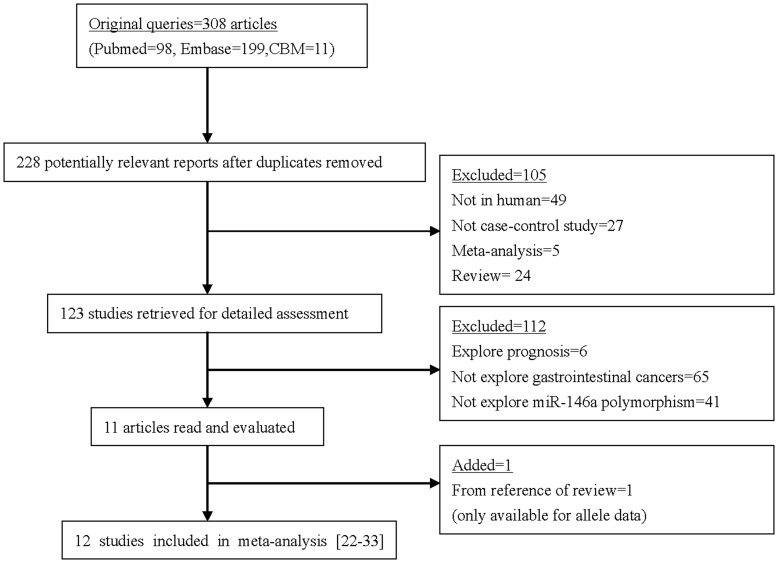
The flow chart of the included studies in the meta-analysis.

Main characteristics of the included publications investigating the association of miR-146a rs2910164 polymorphism with GI cancers are presented in Table 2. There were 308 articles relevant to the searching words (Pubmed:98; Embase:199; CBM:11). The flow chart in Figure 1 summarizes this literature review process. In the current study, a total of 12 eligible studies (4,817 cases and 5,389 controls) met the inclusion criteria [22]–[33].

**Table 2:** Characteristics of studies included in the meta-analysis.*

ID	Study	Year	Ethnic group	Cancer type	Sample size		*P* for HWE	Quality score
					Case	Control		
1	Xiang et al. [22]	2012	Asian	Liver cancer	100	100	0.506	10
2	Min et al. [23]	2011	Asian	Colorectal cancer	446	502	0.443	14
3	Zhou et al. [24]	2011	Asian	Liver cancer	186	483	0.056	14
4	Akkız et al. [25]	2011	Caucasian	Liver cancer	222	222	0.384	13
5	Zhang et al. [26]	2011	Asian	Liver cancer	963	852	0.149	17
6	Okubo et al. [27]	2010	Asian	Gastric cancer	552	697	<0.0001	9
7	Hishida et al. [28]	2010	Asian	Gastric cancer	583	699	0.337	15
8	Guo et al. [29]	2010	Asian	Esophageal cancer	444	468	0.120	14
9	Zeng et al. [30]	2010	Asian	Gastric cancer	304	304	0.122	14
10	Srivastava et al. [31]	2010	Asian	Gallbladder cancer	230	230	0.080	15
11	Xu et al. [32]	2008	Asian	Liver cancer	479	504	0.119	14
12	Ye et al. [33]	2008	Caucasian	Esophageal cancer	346	346	NA	12

* HWE, Hardy-Weinberg equilibrium; NA, not available.

Among the 12 publications, five studies focused on liver cancer [22], [24]–[26], [32], three studies on gastric cancer [27], [28], [30], two studies on esophageal cancer [29], [33], one study on colorectal cancer [23] and one study on gallbladder cancer [31], respectively. Of all studies, ten studies were conducted in Asian populations [22]–[24], [26]–[32], and two in Caucasian populations [25], [33]. The results of Hardy-Weinberg equilibrium test for the distribution of the genotype in control population are shown in Table 2. The genotype frequency distributions of controls in 10 of 12 studies were in agreement with HWE. We could not perform the Hardy-Weinberg equilibrium test for one study, because only the data of allele frequency was available [33]. Thus, for quality assessment, this study was considered as Hardy-Weinberg disequilibrium. Quality scores for the individual studies ranged from 9 to 17, with 83% (10 of 12) of the studies being classified as high quality (≥12).

### Association between miR-146a rs2910164 Polymorphism and GI Cancers

The summary of the meta-analysis for miR-196a2 rs11614913 polymorphism and GI cancers is shown in Table 3. We first analyzed the association in the overall population. Then in order to obtain the exact consequence of the relationship between miR-146a rs2910164 polymorphism and GI cancers susceptibility, stratified analyses by ethnicity, cancer types and quality of studies were performed. When the Q-test of heterogeneity was not significant, we conducted analyses using the fixed effect models. The random effect models were conducted when we detected significant between-study heterogeneity.

**Table 3:** Meta-analysis of miR-146a rs2910164 polymorphism with gastrointestinal cancers.*

Comparisons		Sample size		No. of Studies	Test of association				Test of heterogeneity		
		Case	Control		*OR (95%CI)*	*Z*	*P-value*	*Model*	*χ^2^*	*P-value*	*I^2^(%)*
Overall	G vs C	9634	10778	12	1.07(0.98–1.16)	1.46	0.14	R	21.64	0.03	49.2
	GG+GC vs CC	4471	5043	11	1.14(1.00–1.31)	1.94	0.05	R	18.23	0.05	45.1
	GG vs GC+CC	4471	5043	11	1.06(0.91–1.23)	0.72	0.47	R	20.47	0.03	51.2
	GG vs CC	2394	2767	11	1.17 (0.95–1.44)	1.50	0.13	R	21.91	0.02	54.4
	GC vs CC	3396	3915	11	1.14(1.00–1.31)	1.92	0.05	R	16.25	0.09	38.5
Asian	G vs C	8498	9642	10	1.09(0.99–1.19)	1.70	0.09	R	19.87	0.02	54.7
	GG+GC vs CC	4249	4821	10	1.15(0.99–1.32)	1.88	0.06	R	18.22	0.03	50.6
	GG vs GC+CC	4249	4821	10	1.08(0.91–1.27)	0.89	0.37	R	19.50	0.02	53.9
	GG vs CC	2247	2612	10	1.18(0.95–1.47)	1.48	0.14	R	21.88	0.009	58.9
	GC vs CC	3311	3837	10	1.14(0.99–1.31)	1.82	0.07	R	16.22	0.06	44.5
Caucasian	G vs C	1136	1136	2	0.94(0.77–1.14)	0.65	0.51	F	0.02	0.90	0.0
Liver cancer	G vs C	3824	4298	5	1.07(0.98–1.18)	1.50	0.13	F	5.83	0.21	31.4
	GG+GC vs CC	1912	2149	5	1.11(0.96–1.27)	1.44	0.15	F	3.69	0.45	0.0
	GG vs GC+CC	1912	2149	5	1.08(0.92–1.27)	0.95	0.34	F	7.33	0.12	45.4
	GG vs CC	1015	1147	5	1.18 (0.97–1.43)	1.65	0.10	F	5.76	0.22	30.6
	GC vs CC	1479	1704	5	1.09(0.94–1.26)	1.10	0.27	F	3.28	0.51	0.0
Gastric cancer	G vs C	2878	3400	3	1.05(0.89–1.24)	0.62	0.54	R	4.99	0.08	59.9
	GG+GC vs CC	1439	1700	3	1.15(0.88–1.51)	1.01	0.31	R	6.72	0.03	70.3
	GG vs GC+CC	1439	1700	3	0.93(0.77–1.13)	0.71	0.48	F	4.41	0.11	54.7
	GG vs CC	772	972	3	1.04(0.74–1.47)	0.25	0.81	R	4.98	0.08	59.9
	GC vs CC	1222	1430	3	1.20(0.88–1.64)	1.13	0.26	R	7.77	0.02	74.3
Esophageal cancer	G vs C	1580	1628	2	1.15(0.80–1.66)	0.74	0.46	R	5.26	0.02	81.0
High quality	G vs C	8330	9184	10	1.07(0.97–1.18)	1.31	0.19	R	20.13	0.02	55.3
	GG+GC vs CC	3819	4246	9	1.14(0.96–1.35)	1.52	0.13	R	18.03	0.02	55.6
	GG vs GC+CC	3819	4246	9	1.09(0.94–1.27)	1.15	0.25	R	13.95	0.08	42.6
	GG vs CC	2030	2270	9	1.22(0.97–1.53)	1.67	0.09	R	17.51	0.03	54.3
	GC vs CC	2844	3260	9	1.12(0.95–1.31)	1.35	0.18	R	14.70	0.07	45.6
Low quality	G vs C	1304	1594	2	1.02(0.88–1.19)	0.26	0.79	F	1.18	0.28	15.1
	GG+GC vs CC	652	797	2	1.16(0.94–1.44)	1.41	0. 61	F	0.09	0.77	0.0
	GG vs GC+CC	652	797	2	0.94(0.50–1.76)	0. 19	0. 85	R	3.10	0.08	67.7
	GG vs CC	364	497	2	0.91(0.67–1.23)	0.61	0.54	F	2.07	0.15	51.7
	GC vs CC	552	655	2	1.29(1.02–1.61)	2.16	0.03	F	0.12	0.73	0.0

* OR, odds ratio; vs, versus; R, random effect model; F, fixed effect model.

Overall, when all types of GI cancers were considered together in the meta-analysis, there was no evidence of association between miR-146a rs2910164 polymorphism and the risk of GI cancers in any genetic model (G versus C: OR = 1.07, 95%CI 0.98−1.16, *P* = 0.14; GG+GC versus CC: OR = 1.14, 95%CI 1.00−1.31, *P* = 0.05; GG versus GC+CC: OR = 1.06, 95%CI 0.91−1.23, *P* = 0.47; GG versus CC: OR = 1.17, 95%CI 0.95−1.44, *P* = 0.13; GC versus CC: OR = 1.14, 95%CI 1.00−1.31, *P* = 0.05).

Next, we performed subgroup analyses according to ethnicity of the studies. In Asians, no significant association between miR-146a rs2910164 polymorphism and the risk of GI cancers was found for all genetic model (G versus C: OR = 1.09, 95%CI 0.99−1.19, *P* = 0.09; GG+GC versus CC: OR = 1.15, 95%CI 0.99−1.32, *P* = 0.06; GG versus GC+CC: OR = 1.08, 95%CI 0.91−1.27, *P* = 0.37; GG versus CC: OR = 1.18, 95%CI 0.95−1.47, *P* = 0.14; GC versus CC: OR = 1.14, 95%CI 0.99−1.31, *P* = 0.07). In Caucasians, there was no evidence of association between the variant genotypes of miR-146a rs2910164 polymorphism and the risk of GI cancers in the allelic comparison (G versus C: OR = 0.94, 95%CI 0.77−1.14, *P* = 0.51).

Moreover, we investigated the effect of the miR-146a rs2910164 polymorphism on the susceptibility to subtypes of GI cancers. No evidence of association was observed in any genetic model between miR-146a rs2910164 polymorphism and risk of liver cancer (G versus C: OR = 1.07, 95%CI 0.98−1.18, *P* = 0.13; GG+GC versus CC: OR = 1.11, 95%CI 0.96−1.27, *P* = 0.15; GG versus GC+CC: OR = 1.08, 95%CI 0.92−1.27, *P* = 0.34; GG versus CC: OR = 1.18, 95%CI 0.97−1.43, *P* = 0.10; GC versus CC: OR = 1.09, 95%CI 0.94−1.26, *P* = 0.27), gastric cancer (G versus C: OR = 1.05, 95%CI 0.89−1.24, *P* = 0.54; GG+GC versus CC: OR = 1.15, 95%CI 0.88−1.51, *P* = 0.31; GG versus GC+CC: OR = 0.93, 95%CI 0.77−1.13, *P* = 0.48; GG versus CC: OR = 1.04, 95%CI 0.74−1.47, *P* = 0.81; GC versus CC: OR = 1.20, 95%CI 0.88−1.64, *P* = 0.26) and esophageal cancer (G versus C: OR = 1.15, 95%CI 0.80−1.66, *P* = 0.46).

We also performed subgroup analysis according to quality of studies. In the subgroup of high quality studies, no significant association between miR-146a rs2910164 polymorphism and risk of GI cancers was observed (G versus C: OR = 1.07, 95%CI 0.97−1.18, *P* = 0.19; GG+GC versus CC: OR = 1.14, 95%CI 0.96−1.35, *P* = 0.13; GG versus GC+CC: OR = 1.09, 95%CI 0.94−1.27, *P* = 0.25; GG versus CC: OR = 1.22, 95%CI 0.97−1.53, *P* = 0.09; GC versus CC: OR = 1.12, 95%CI 0.95−1.31, *P* = 0.18). In the subgroup of low quality studies, there was also no evidence of association between miR-146a rs2910164 polymorphism and the risk of GI cancers in any genetic model (G versus C: OR = 1.02, 95%CI 0.88−1.19, *P* = 0.79; GG+GC versus CC: OR = 1.16, 95%CI 0.94−1.44, *P* = 0.61; GG versus GC+CC: OR = 0.94, 95%CI 0.50−1.76, *P* = 0.85; GG versus CC: OR = 0.91, 95%CI 0.67−1.23, *P* = 0.54; GC versus CC: OR = 1.29, 95%CI 1.02−1.61, *P* = 0.03).

### Evaluation of Publication Bias (Table 4)

The results of Egger’s linear regression test are shown in Table 4. The shape of the funnel plots did not reveal any evidence of obvious asymmetry for all genetic models in the overall meta-analysis. Egger’s test was used to provide statistical evidence of funnel plot symmetry. The intercept *a* provides a measure of asymmetry, and the larger its deviation from zero the more pronounced the asymmetry. The results still did not present any obvious evidence of publication bias for any of the genetic models. Egger’s test only detected evidence of publication bias in the subgroup analysis of gastric cancer for allelic contrast (P = 0.004). However, Egger’s test was not applied in some comparisons due to the small number of studies.

**Table 4:** Egger’s linear regression test to measure the funnel plot asymmetric.*

Groups	Y axis intercept: *a (95%CI)*				
	G vs C	GG+GC vs CC	GG vs GC + CC	GG vs CC	GC vs CC
Overall	0.02 (−3.33−3.37)	0.29(−2.02−2.60)	0.80(−3.57−5.17)	0.75(−2.24−3.74)	0.02(−2.19−2.23)
Asian	0.85(−3.34−5.05)	0.40(−2.47−3.28)	1.07(−3.59−5.72)	1.06(−2.58−4.70)	−0.06(−2.81–2.70)
Liver cancer	0.24(−5.20−5.67)	−0.01(−3.56−3.55)	2.18(−4.03−8.38)	0.90(−3.81−5.60)	−0.20 (−3.53−3.13)
Gastric cancer	8.70(7.98–9.43)	7.20(−53.24–67.63)	7.63(−88.45–103.70)	9.47(−18.33–37.26)	6.26(−80.96–93.49)
High quality	−0.81(−5.23–3.61)	0.25(−2.78–3.26)	−0.09(−5.72–5.54)	0.25(−3.33–3.84)	0.16(−2.60–2.91)

* vs, versus.

## Discussion

In the present meta-analysis with 4,817 cases and 5,389 controls, there was no evidence of association between miR-146a rs2910164 polymorphism and the risk of GI cancers. Similar results were found in the subgroup analyses by ethnicity, cancer types, and quality of studies. The current study is the largest meta-analysis of the association between miR-146a rs2910164 polymorphism and the risk of GI cancers.

In 2011, several meta-analyses were conducted to investigate the association between miR-146a rs2910164 polymorphism and overall cancer risk. Xu et al. [42] and Qiu et al. [43] both identified that miR-146a rs2910164 polymorphism was not associated with overall cancer risk. In another meta-analysis, overall increased cancer risk was only found in dominant model (*P* = 0.02) [44]. Nevertheless, the authors did not adjust the significance alpha level. If multiple comparisons were corrected, a negative result would be obtained. In consistent with these reports, we did not find any association between miR-146a rs2910164 polymorphism and the risk of GI cancers in overall analysis. When stratified by cancer types, Xu et al. [42] found that the C allele of miR-146a rs2910164 polymorphism might be associated with protection from digestive cancer in subgroup analysis. But the subgroup analysis only included three studies [29], [31], [32]. Contrary to the result, our meta-analysis provided more sufficient evidence that miR-146a rs2910164 was not a functional polymorphism on GI cancers susceptibility based on larger sample sizes and increased statistical power. Similarly, in subgroup analysis, we consistently showed no association between this SNP and GI cancers.

The current meta-analysis is based on a single polymorphism strategy to explore the association between miR-146a gene polymorphism and GI cancers. Duan and colleagues have identified 323 SNPs in 227 human known miRNAs [45]. Though a single SNP has limited effect on the risk of GI cancers, interactions of multiple SNPs in miRNA-related genes might augment the effect. Development of GI cancers is a multistage process, and a single polymorphism might have a limited impact on GI cancers susceptibility [10]. More comprehensive haplotype-based or multiple polymorphisms-based strategies rather than a single polymorphism-based strategy are warranted, which may provide more precise information on genetic contribution of miR-146a gene polymorphism to GI cancers etiology. In addition to genetic predisposition, environmental exposure, such as smoking, alcohol consumption, and diet, is also thought to play a crucial role in the etiology of GI cancers [46]. Gene-environment interactions should be considered in further studies if individual data of environmental exposure are available.

Certain potential limitations exist in our meta-analysis. Firstly, the controls for one study included in this meta-analysis were not in Hardy-Weinberg equilibrium. To some extent, the results of genetic association studies might be distorted. Secondly, publication bias was detected in the subgroup analysis of gastric cancer for allelic contrast. This may also distort the meta-analysis. Thirdly, as with most meta-analyses, results should be interpreted with caution because of obvious between-study heterogeneity in some comparisons. Fourthly, if individual data were available, we could perform a more precise analysis with an adjustment estimate. Finally, colorectal cancer is the commonest cancer of the alimentary tract. Lacking sufficient eligible studies on colorectal cancer limited our further stratified analyses.

In conclusion, results from meta-analysis of published data show that miR-146a rs2910164 polymorphism is not associated with GI cancers susceptibility. It is necessary to conduct more well-designed studies based on larger sample sizes and homogeneous cancer patients.

## Supporting Information

Table S1 **Checklist of items to include in this meta-analysis.**
(DOC)
